# Coping strategies and symptoms of Adjustment Disorder among adults with Attention Deficit Hyperactivity Disorder (ADHD) during the Covid-19 pandemic

**DOI:** 10.1371/journal.pone.0309082

**Published:** 2024-08-19

**Authors:** Katarina Danielsson, Filip K. Arnberg, Kristina Bondjers

**Affiliations:** 1 Department of Medical Sciences, Psychiatry, Uppsala University, Uppsala, Sweden; 2 Department of Medical Sciences, National Centre for Disaster Psychiatry, Uppsala University, Uppsala, Sweden; 3 National Centre for Violence and Traumatic Stress Studies, Oslo, Norway; University of Catania Libraries and Documentation Centre: Universita degli Studi di Catania, ITALY

## Abstract

**Background:**

The current study examined whether coping strategies and symptoms of Adjustment Disorder in adults with ADHD differed from what was observed in the general adult population during the Covid-19 pandemic, and compared the extent to which coping strategies and symptom levels of Adjustment Disorder were related to ADHD.

**Method:**

This cross-sectional study was based on survey data collected during the spring of 2021 from 231 adult ADHD patients in specialist care and 1148 volunteers without ADHD in Sweden. The survey included questions about sociodemographic and clinical characteristics, along with the Brief-COPE and Adjustment Disorder–New Module 8 questionnaires. Regression models adjusting for sociodemographic and clinical characteristics were used for between-group comparisons of coping strategies and symptoms of Adjustment Disorder.

**Results:**

There were some notable differences in the use of coping strategies between persons with and without ADHD; however, many of these differences were not observed in the adjusted models. The use of behavioral disengagement was more frequently observed among individuals with ADHD, whereas planning was more common among individuals without ADHD. Individuals with ADHD appeared to show higher symptom levels of Adjustment Disorder during the pandemic. Passive coping strategies, such as denial, self-blame, and behavioral disengagement, were associated with higher symptom levels of adjustment disorder in both individuals with and without ADHD.

**Conclusion:**

In conclusion, the results highlight that persons with ADHD may need more support to adjust to large societal changes than the general public. Potential targets for intervention towards members of this group include reducing resignation and maladaptive coping strategies.

## Introduction

Attention Deficit Hyperactivity Disorder (ADHD) is a common neurodevelopmental disorder in children and adults. The prevalence is around 7% in children and 2–3% in adults. The symptoms of this disorder include inattention, impulsivity, hyperactivity, and distractibility. Moreover, the disorder is often accompanied by psychological and physiological comorbidities, and causes functional impairment in different domains in life [1, 2]. Even with treatment, whether pharmacological or non-pharmacological, individuals with ADHD can still experience functional impairment and be more sensitive to stress. Previous research has demonstrated that subjectively perceived stress is associated with ADHD and may elevate the risk for comorbid disorders and further impairments [3]. Furthermore, the current evidence suggests that adults with ADHD who are exposed to stressful events rate subjective stress higher, as well as experience a stronger release of stress hormones, than adults without ADHD [4, 5]. There is also evidence that the psychological distress experienced by persons with ADHD are linked to problematic use of internet, which also is a common feature in this group [6, 7]

Humans employ various coping strategies to deal with stressful events [8]. These strategies are often categorized into active and passive approaches. According to many studies, active coping strategies are considered to decrease harmful forms of stress and impart positive effects, such as increased mental well-being, whereas passive coping strategies can exacerbate existing anxiety and are associated with further impairments to quality-of-life [9–12].

The Covid-19 pandemic, which was most pronounced between 2020–2023, was a highly stressful event for many people. Significant restrictive measures were rapidly introduced and implemented to limit the spread of infection. For instance, the Swedish government implemented non-compelling recommendations that limited the availability of cultural, sports, and other social events. As such, universities implemented remote classes, citizens were encouraged to stay home with minor symptoms or when sick, workplaces were encouraged to use remote work whenever possible, and visits to older relatives at retirement homes were prohibited [13]. Even though these were recommendations, which meant that they were not as strictly enforced as in other countries, high adherence to these recommendations nevertheless had significant effects on the Swedish population’s social and workplace behavior. An increased level of depressive, anxiety, and insomnia symptoms were observed among the Swedish population during the peak years of the pandemic [14, 15]. Studies in different countries in healthy populations and in populations with different diagnoses has demonstrated that the pandemic was related to difficulties to reach healthcare, inadequate patient support, ineffective self-care, and an increase in problematic internet use [16–19]. Further on, social support affect self-management positively [20]. Because of the societal restrictions put in place, it is likely that there was less availability of social support during the Covid-19 pandemic. Additionally, both children with mental health problems, such as ADHD, and their parents, were negatively affected by the Covid-19 pandemic [21–23].

In addition to the aforementioned problems, it has been suggested that Adjustment Disorder (AjD) was a common response to the stressors related to the pandemic, and this trend was prevalent among both the general population and vulnerable individuals [24]. AjD is defined as a maladaptive reaction to a stressful life event, such as job strain, economic difficulties, relationship problems, or severe life changes [25]. A pan-European study of the general population found that the self-reported rate of probable AjD was 18.2% in the summer of 2020; [26]. Higher AjD symptom levels were found among individuals with a current or previous diagnosis of a mental health disorder, a finding that has also been confirmed in other samples [27]. Pre-pandemic research indicates that maladaptive coping styles, such as passive, self-blaming, and/or avoidant strategies, are more prevalent among individuals with AjD [28]. Also, problematic internet use is more common among persons with ADHD [6, 7]. As such, it is plausible that individuals with ADHD have been more affected during the challenging times of the past few years because these individuals often heavily depend on routines to manage in life. Furthermore, some individuals with ADHD are highly vulnerable to stress and/or present with severe functional impairments that require ongoing, stable social services [29, 30]. According to a Israeli study that was conducted during the pandemic, increased symptoms of ADHD were associated with lower levels of adherence to preventive measures, such as focusing on social distancing, personal hygiene measures, and wearing a mask [31]. Furthermore, several studies on adolescents and children with ADHD revealed difficulties in maintaining routines, use of maladaptive coping (e.g., substance use), and the worsening of ADHD symptoms during the pandemic [32, 33]. In addition, a couple of studies that focused on the use of coping strategies among persons with ADHD reported an increase in maladaptive strategies which exceeded what was observed in the healthy population [34, 35]. To better understand how to help individuals with ADHD adjust to societal stressors such as a pandemic, the current study aims to examine how coping strategies and symptoms of AjD among adults with ADHD differed from the general adult population during the Covid-19 pandemic. Our hypothesis was that persons with ADHD would be more prone to use maladaptive coping strategies and have more symptoms of adjustment disorder than the general population.

A secondary aim was to investigate how different coping strategies were associated with AjD symptom levels in persons with ADHD and the general population.

## Methods

### Design and participants

The data analyzed in this study originate from two populations: individuals with a diagnosis of ADHD; and a general population sample extracted from the Swedish cohort of the European Society for Traumatic stress ADJUST study, a pan-European collaboration [36]. Eligible participants were at least 18 years of age and reported primary residence in Sweden from 10th of March 2020. There were no further exclusion criteria. Individuals with ADHD were mainly recruited via the Neuropsychiatric Department at Uppsala University Hospital, Sweden. All patients with ADHD who attended the clinic were invited to participate via participation forms sent from the hospital’s online platform. No exclusion criteria were employed. No power analysis was conducted for this group. A total of 1363 participation forms were distributed, with 390 patients providing their written informed consent to participate, and 226 patients ultimately providing a response. The questionnaire was answered from 16^th^ of February 2021 until the 29^th^ of July 2021.

The Swedish ADJUST study was a longitudinal online survey that included four assessment points between July 7, 2020 and August 18, 2021. A majority of the sample was self-recruited via social media, and a smaller proportion (24.7%) was recruited using postal invitations to a randomly-selected sample of the Swedish adult population. A written informed consent was obtained from the participants before continuing to the questionnaire. To enable comparison between individuals participating in the Swedish ADJUST study and the participants recruited from the neuropsychiatric department, only data from the third assessment point (January 3, 2021 to May 25, 2021) in the ADJUST study were used (n = 1805). A priori power analysis was conducted for this sample, according to which 1000 participants or above would yield adequate power for detecting weak associations in multiple regression analysis.

The participants were between 18 and 65 years of age. As the average age values of the two recruitment groups noticeably differed, participants 65 years of age or older (n = 268) were excluded from the ADJUST sample. Furthermore, participants with missing data on any of the questions included in the current analysis were excluded (n = 379). Some of the participants in the ADJUST study reported having an ADHD diagnosis (n = 10), and were thus included in the ADHD group for the current analysis. The final sample comprised 1148 participants without a diagnosis of ADHD and 231 participants with a diagnosis of ADHD. The Swedish ethical review authority approved this study (DNR.2020-05058 and 2020–03217).

### Measures

#### Sociodemographic data

The questionnaire included items covering sociodemographic information, including self-reported age, gender, working status, relationship status, and whether or not the person had any friends. Working status was dichotomized into: (1) attending educational activities or work; and (2) no educational activities or work, or being on full-time sick leave. Relationship status was dichotomized: into (1) being in a relationship; and (2) not being in a relationship. Answers to the question about having friends were dichotomized into: (1) yes; and (2) no.

#### Clinical data

For the measure of self-rated health (SRH), participants were asked to evaluate their health status by responding to the question “How is your health?” using a five-point Likert scale ranging from very good (1) to very poor (5). To assess the prevalence of Covid-19 infection among the participants, they were asked whether they had been infected at any time prior to the survey (yes/no). The Patient Health Questionnaire-4 item version (PHQ-4) was used to screen for symptoms of depression and anxiety [37] by having respondents rate how frequently they have been bothered by the two core symptoms of depression and anxiety, respectively, during the past two weeks. The respondents provided answers using a four-point scale ranging from none at all (0) to almost every day (3). A total score was then obtained by summating the scores across all of the items.

#### Adjustment Disorder (AjD)

Symptoms of AjD were assessed using the Adjustment Disorder–New Module 8 scale (ADNM-8) [38]. The ADNM-8 consists of eight items, and respondents were asked to indicate the most stressful experience of the pandemic and then–while keeping this situation in mind–rate how often they had been bothered by each symptom on a 4-point Likert scale (1 = never, 2 = rarely, 3 = sometimes, 4 = often). Previous research has presented results that support the validity and reliability of ADNM-8, which has been suggested to have adequate psychometric properties [39].

#### Coping strategies

Participants’ use of coping strategies was assessed with the Brief-COPE instrument, which measures 14 types of coping strategies (acceptance, active coping, using emotional support, using instrumental support, positive reframing, planning, humor, denial, substance use, behavioral disengagement, venting, self-distraction, religion, and self-blame). Respondents were asked to rate 28 behaviors using a four-point Likert scale ranging from “I have not done this at all” (0) to “I have been doing this a lot” (3) [40].

### Data analysis

The data analysis included several steps. First, we calculated proportions and mean scores for demographical variables (i.e., age, gender, work-status, relationship status, and having friends), clinical data (self-rated health, Covid-19 infection and PHQ-4), and the ADNM-8 and Brief-COPE instruments for all participants, as well as separately for individuals with and without ADHD. Independent samples t-tests were used to compare mean values, while the Chi^2^ test of independence was used to discern between-group differences in dichotomized variables. We then ran several multiple linear regression analyses. First, we used a stepwise regression model to analyze each coping strategy as an outcome, with only ADHD as a predictor (model 1); the model was then adjusted for sociodemographic data (model 2) and sociodemographic and clinical (depression, anxiety, self-reported health) data (model 3) as confounders. Second, we regressed AjD symptoms on ADHD status, adjusting for sociodemographic and clinical data. Finally, we performed a regression, stratified for ADHD status or not, of symptom levels of AjD as outcome against coping strategies as predictors while adjusting for sociodemographic and clinical data as confounders. All of the data analyses were performed in SPSS Statistics software (version 26.0; IBM SPSS, Chicago, IL, USA). There were missing data in about 18,6% of the sample. Complete case analysis was used in all calculations. Although there are more sophisticated methods for addressing missing data, we chose to use complete case analysis as this was cross sectional study and for most of the cases whole sets of, or several, questionnaires were missing.

## Results

### Sociodemographic and clinical characteristics of participants

Sociodemographic and clinical characteristics, along with the ADNM-8 scores, of the total sample and stratified by ADHD status, are shown in Table 1. A larger proportion of respondents without ADHD reported that they were working, living in a relationship, and had friends in comparison to respondents with ADHD. The individuals with ADHD were younger and more likely to be male when compared to individuals without ADHD. Furthermore, individuals with ADHD gave lower ratings of their health status and had higher symptom levels of depression, anxiety, and AjD than individuals without ADHD. The two groups did not significantly differ in rates of Covid-19 infection.

**Table 1:** Sociodemographic and clinical characteristics, along with symptoms of AjD, in participants with and without ADHD.

	Total (n = 1379)	ADHD (n = 231)	Non-ADHD (n = 1148)	ADHD versus Non-ADHD
Age (Mean±SD)	46.81±11.13	35.59±11.78	49.07±9.51	*t* = -16.35, *p<0*.*001*
Female gender	75.1%	64.5%	77.3%	*Χ*^2^ = 22.30, *p<0*.*001*
Working/studying	85.6%	78.8%	87.0%	*Χ*^2^ = 10.60, *p<0*.*01*
Having friends	88.9%	83.1%	90.0%	*Χ*^2^ = 9.42, *p<0*.*01*
Living in a relationship	62.1%	39.4%	66.6%	*Χ*^2^ = 60.63, *p<0*.*001*
Covid-19 infection	11.7%	12.6%	11.6%	*Χ*^2^ = 0.17, *p = 0*.*37*
SRH (mean±SD)	2.29±0.98	2.76±1.02	2.21±0.94	*t* = 8.37, *p<0*.*001*
PHQ 4 (mean±SD)	3.83±3.46	5.59±3.26	3.47±3.38	*t* = 8.75, *p<0*.*001*
ADNM-8 (mean±SD)	16.35±6.75	20.52±6.59	15.51±6.46	t = 10.572, *p<0*.*001*

*SRH =* Self-rated health, *PHQ 4 =* Patient Health Questionnaire-4 item version, *ADNM-8* = Adjustment Disorder–New Module 8 scale

### Use of coping strategies

The individuals with ADHD reported more frequent use of coping strategies like denial, substance use, behavioral disengagement, self-distraction, venting, self-blame, and informational support when compared to respondents without ADHD, with the latter group reporting higher use of coping strategies like acceptance and positive remodeling, see Table 2. As for the regression analysis, when no variables were adjusted for, ADHD was positively associated with use of the coping strategies instrumental support, denial, venting, self-blame, self-distraction, and behavioral disengagement. After adjusting for demographic data, coping strategies of venting, self-blame and behavioral disengagement continued to be more associated with the ADHD group, whereas acceptance as a coping strategy continued to be more associated with the non-ADHD group (Model 2, Table 3). However, when the regression model was also adjusted for clinical variables, only behavioral disengagement remained associated with the ADHD group and planning became associated with the non-ADHD group (Model 3, Table 3). Detailed data is shown in S1 Table.

**Table 2:** Comparison of coping strategies in the ADHD and non-ADHD groups.

Coping strategy (score; mean±SD)	Total	ADHD	Non-ADHD	ADHD vs Non-ADHD
Active coping	1.57±0.87	1.55±0.84	1.58±0.88	t = -0.39 p = 0.7
Acceptance	1.03±0.80	2.04±0.82	2.20±0.73	*t* = -2.92, *p<0*.*01*
Planning	1.61±0.83	1.60±0.85	1.61±0.83	t = -0.09, p = 0.93
Positive reframing	1.37±0.85	1.27±0.84	1.39±0.85	*t* = -1.98, *p<0*.*05*
Humor	1.24±0.94	1.29±0.98	1.23±0.93	t = 0.79. p = 0.4
Instrumental support	1.03±0.80	1.18±0.84	1.00±0.80	*t* = 3.11, *p<0*.*01*
Emotional support	1.28±0.85	1.35±0.89	1.27±0.84	t = 1.30, p = 0.20
Denial	0.18±0.42	0.24±0.49	0.17±0.40	*t* = 2.04, *p<0*.*05*
Venting	1.28±0.85	1.39±0.88	1.26±0.84	t = 2.19, *p<0*.*05*
Self-blame	0.66±0.76	0.97±0.91	0.60±0.71	*t* = 5.98, *p<0*.*001*
Substance use	0.20±0.51	0.26±0.56	0.19±0.49	*t* = 1.75, *p = 0*.*06*
Behavioral disengagement	0.28±0.56	0.54±0.67	0.23±0.52	*t* = 6.67, *p<0*.*001*
Self-distraction	1.07±0.84	1.25±0.84	1.03±0.83	*t* = 3.37, *p<0*.*001*
Religion	0.45±0.79	0.38±0.72	0.46±0.80	t = -1.57, p = 0.12

**Table 3:** Associations between different coping strategies and ADHD diagnosis in unadjusted models (Model 1), models adjusting for sociodemographic data (Model 2) and both sociodemographic and clinical data (Model 3).

Coping strategy								
		Active coping	Acceptance	Planning	Positive reframing	Humor	Instrumental support	Emotional support
Model 1: ADHD	B	-0.024	-0.157**	-0.005	-0.121*	0.054	0.039**	0.079
	SE	0.063	0.054	0.060	0.025	0.068	0.013	0.061
Model 2: ADHD +	B	-0.002	-0.139*	-0.081	-0.283	-0.119	0.022	0.057
Sociodem.	SE	0.072	0.062	0.068	0.052	0.078	0.011	0.068
Model 3: ADHD +	B	-0.025	-0.092	-0.148*	-0.051	-0.112	0.011	0.034
Sociodem. + clin.	SE	0.072	0.062	0.067	0.071	0.079	0.011	0.069
		**Denial**	**Venting**	**Self-blame**	**Self-distraction**	**Substance use**	**Behavioral disengagement**	**Religion**
Model 1: ADHD	B	0.070*	0.133*	0.377***	0.226***	0.069	0.313***	-0.083
	SE	0.030	0.061	0.054	0.060	0.036	0.040	0.057
Model 2: ADHD +	B	0.049	0.164*	0.133*	0.119	0.068	0.186***	-0.053
Sociodem.	SE	0.035	0.068	0.060	0.068	0.042	0.045	0.065
Model 3: ADHD +	B	0.022	0.092	-0.011	0.037	0.034	0.102*	-0.064
Sociodem. + clin.	SE	0.034	0.067	0.053	0.066	0.041	0.041	0.066

Note: The models were run separately for each coping strategy. Model 1 included only ADHD status as a predictor. Model 2 was the same as Model 1, but also adjusted for sociodemographic data (age, gender, working status, relationship status, friends), while Model 3 was adjusted for both sociodemographic and clinical data (Covid-19 infection, self-rated health, and depressive and anxiety symptoms).

* = p<0.05

** = p<0.01

*** = p<0.001

### Symptoms of AjD and coping strategies

To examine the relationships between ADHD-status, coping strategies, and symptoms of AjD, we first conducted a regression using ADHD status as predictor of symptoms of AjD. The results showed that having ADHD was associated with higher ADNM-8 scores when compared to individuals without ADHD (B = 5.009, SE = 0.467, β = 0.277, p = 0.0001, CI 95% = 4.092–5.926, R^2^ = 0.077). Individuals with ADHD still demonstrated higher ADNM-8 scores even when adjusting for sociodemographic data (B = 4.409, SE = 0.528, β = 0.244, p = 0.0001, CI 95% = 3.374–5.445, R^2^ = 0.128), as was the case when the regression was adjusted also for clinical data (B = 2.930, SE = 0.429, β = 0.162 p = 0.0001, CI 95% = 2.089–3.771, R^2^ = 0.440).

We then conducted a regression analysis to assess differences in the relationships between AjD symptoms and coping strategies in the ADHD and non-ADHD groups; these regressions were adjusted for sociodemographic and clinical data (Table 4). The associations between coping strategies and AjD symptoms were generally similar across both the ADHD and non-ADHD groups; more specifically, denial, self-blame, and behavioral disengagement demonstrated the strongest associations with symptoms of AjD in both groups. Positive reframing, humor, and religion were not significantly associated symptoms of AjD in either group. Furthermore, lower levels of acceptance and higher levels of substance use were associated with symptoms of AjD in the non-ADHD group, while this pattern was not observed in the ADHD group, see Table. Detailed data is shown in S2 Table.

**Table 4:** Associations between different coping strategies and symptoms of AjD in adults with and without ADHD.

	ADHD		Non-ADHD	
Coping strategy	B	SE	B	SE
Active coping	1.81**	0.42	1.44**	0.17
Acceptance	0.12	0.45	-0.44*	0.20
Planning	2.05**	0.42	1.45**	0.18
Positive reframing	0.30	0.44	0.03	0.18
Humor	0.18	0.37	-0.27	0.16
Instrumental support	1.95**	0.44	1.20**	0.19
Emotional support	1.88**	0.40	0.87**	0.18
Denial	2.63**	0.73	2.56**	0.38
Venting	2.07**	0.42	1.42**	0.18
Self-blame	1.93**	0.44	1.82**	0.25
Self-distraction	2.3**	0.40	1.70***	0.19
Substance use	0.75	0.67	1.16**	0.31
Behavioral disengagement	2.62**	0.56	1.81**	0.32
Religion	0.45	0.50	0.19	0.19

The values are adjusted for sociodemographic and clinical data.

* = p<0.05

** = p<0.01

*** = p<0.001

## Discussion

There are four main findings from the current study. First, while some differences in the use of coping strategies between persons with and without ADHD were observed, many of these between-group differences nevertheless appear attributable to sociodemographic (gender, age, working status, and relationship status) and clinical (Covid-19 infection, self-rated health, and depressive and anxiety symptoms) data. Second, in terms of coping strategies, the use of behavioral disengagement was more evident among individuals with ADHD, whereas planning was more common among individuals without ADHD. Third, individuals with ADHD appear to have suffered more from AjD symptoms during the pandemic than the general population. Fourth, passive coping strategies, specifically denial, self-blame, and behavioral disengagement, were associated with AjD symptoms in both individuals with and without ADHD,

This study furthers our understanding of which coping strategies persons with ADHD rely on during stressful life events, and also highlights that some strategies appear to be less beneficial than others. For example, the group of individuals with ADHD analyzed in this study used more passive coping strategies than those without ADHD, and this dynamic remained significant even after adjusting for sociodemographic and clinical data. Moreover, a positive association between passive coping strategies and symptoms of AjD was observed. Behavioral disengagement, denial and venting were more evident in the ADHD group than the sample of the general population. However, when adjusting the regression for depression, anxiety, and self-reported health, the significant between-group differences in the prevalence of denial and venting were no longer evident. These results are in line with previous research on coping strategies among ADHD populations [34, 35]. For instance, prior research has shown that persons with ADHD are more prone to use maladaptive coping strategies than the general population; as such, individuals with ADHD are likely to aggressively confront stressful situations, attempt to avoid and escape these situations, react emotionally to stress, and feel preoccupied with problems [35, 41, 42]. Maladaptive coping strategies have been linked to higher levels of depression, while maladaptive emotional regulation strategies have been linked to anxiety [43, 44]. On the other hand, as this is a cross-sectional study, it is important to underscore that the results cannot make directional claims between coping or AjD.

Previous research has indicated that use of maladaptive coping strategies of denial, substance use, venting, and behavioral disengagement, among first year bachelor students, was associated with increased levels of anxiety and depression during the Covid-19 pandemic [45]. In our results some of the maladaptive strategies could be explained by higher levels of depressive and anxiety symptoms in the ADHD group, since some of the maladaptive copingstrategies were no longer evident after adjusting for clinical data. This could indicate that persons with ADHD and comorbid depression or anxiety may be more likely to use maladaptive coping strategies, or that maladaptive coping strategies will likely worsen an individual’s depressive and anxiety symptoms. In the present study, the adaptive coping strategy of acceptance was more prevalent in the non-ADHD group than the ADHD group, although this association did not hold when the regression adjusted for clinical data, suggesting that the clinical characteristics that we adjusted for in this study have a higher impact on the use of acceptance as compared to ADHD status. Beneficial coping strategies during the Covid-19 pandemic have been studied among nurse students and families: Positive attitude towards the stressful situation, social support and religion were protective factors [46, 47]. However, in a study among healthcare personnel, positive attitude was the main protective coping strategy whereas religion was not found to be a beneficial coping strategy [48]. It might be helpful for persons with ADHD to use these coping strategies more frequently, however a person’s social context seems to play a role in which coping strategies to choose. Longitudinal studies are warranted to better understand which strategies should be used among persons with ADHD. It has been established that persons with ADHD experience more stress and have more difficulties adjusting to new situations [4, 5]. For example, a recent study found that students with ADHD have more difficulties adjusting to the college setting, along with lower levels of self-reported social skills and self-esteem relative to peers without an ADHD diagnosis [49].Furthermore, persons with ADHD have a tendency to use maladaptive coping strategies more frequently than the general population. It could be hypothesized that these dynamics are connected to risk of developing AjD, which would explain why individuals with ADHD experience more symptoms of AjD than the general population in the present study.

Several of the coping strategies, both adaptive and maladaptive, were associated with higher AjD symptom levels. This link is unsurprising, as difficulties in adjusting and perceived stress are often connected. It is not always bad to feel stress, and some stress can be positive because it forces an individual to take action by, for example, planning, seeking informational and emotional support, and trying various alternative solutions to a problem. Nevertheless, adjustment difficulties and stress can also paralyze an individual, effectively worsening both the situation and well-being [50]. This partly explains why self-blame, venting, and behavioral disengagement showed stronger associations with symptoms of AjD in both groups even though several coping strategies, both adaptive and maladaptive, were associated with AjD symptoms. At the end, only two between-group differences were found, namely, increased substance use and less acceptance was associated with AjD symptoms in the non-ADHD group.

### Methodological considerations and future directions

Coping strategies and an individual’s capacity to adjust to a stressful situation are influenced by gender, age, social status and health status [49]. A methodological consideration of the present study is that the two analyzed groups differed noticeably in terms of both sociodemographic and clinical data. This is why the performed regression analyses were adjusted for these factors, although it is important to state that statistical adjustment may not fully remove the bias stemming from between-group differences. However, it is also important to keep in mind that the ADHD group has a fair degree of functional impairment and, as such, it is not surprising that the sociodemographic and clinical data of this group differed from that of the general population sample. A non-ADHD comparison group that was matched on functional impairment or other forms of clinical data would likely introduce other risks of bias. Another noteworthy consideration is that both groups involved a large share of women. Considering that men use avoidant coping strategies more frequently than women, and the prevalence of ADHD is higher among men [51, 52] care should be exercised when generalizing these findings to male populations. Nevertheless, the gender distribution may be less of a threat to the internal validity of the study as the proportion of women was similar in both groups. It is also important to consider national differences in the governmental restrictions that were implemented during Covid-19 when generalizing the results from the Swedish population to international contexts.

As for the study design, we analyzed data from self-reported and brief questionnaires, which involve the risk of subjective bias, e.g., under- or overreporting. Furthermore, there might be differences between those who choose to participate in a survey and those who do not–this form of bias is often difficult to avoid. Missing data is also a problem in clinical science. It reduces sample size and statistical power. It can increase the uncertainty of the estimated parameters. Furthermore, it can increase bias and complicate the analysis of the study. Complete case analysis was adapted to handle missing data in the present study, since this was not a longitudinal study and for most of the cases hole sets of, or several, questionnaires were missing. A notable strength of the study is that almost all of the participants (95%) in the ADHD group had a clinical diagnosis of ADHD and were being treated at the out-patient clinic; this group thus represents a clinically relevant sample. Finally, we again note that the study was cross-sectional, which precludes any conclusions about directionality between coping strategies and symptoms of AjD. Future studies could apply a longitudinal design to observe how AjD and coping strategies develop over time among persons with ADHD.

## Conclusion

In conclusion, the results revealed that both the ADHD and non-ADHD groups used active coping strategies. However, having ADHD was associated with more pronounced AjD symptom levels and an increased likelihood of using maladaptive coping strategies. In light of the caveats noted above, the results suggest that persons with ADHD may need more help than other members of the population to adjust to large societal changes, such as a pandemic. Potential targets for intervention towards members of this group include reducing resignation and maladaptive coping strategies among individuals with ADHD.

## Supporting information

S1 Table Detailed information about the associations between different coping strategies and ADHD diagnosis. (DOCX)

S2 Table Detailed information about the associations between different coping strategies and symptoms of AjD in adults with and without ADHD. (DOCX)
