# Supplementary material for: Coping strategies and symptoms of Adjustment Disorder among adults with Attention Deficit Hyperactivity Disorder (ADHD) during the Covid-19 pandemic
Source: PLoS One. 2024 Aug 19;19(8):e0309082. doi: 10.1371/journal.pone.0309082 (PMC11332942; doi:10.1371/journal.pone.0309082)
Supplement: S1 Table — (DOCX) [file pone.0309082.s001.docx]

**S1 Table** **1** **Detailed information about the association between different coping strategies and ADHD diagnosis.**

|  |  |  | **Coping strategy** | | | | | | |
| --- | --- | --- | --- | --- | --- | --- | --- | --- | --- |
|  |  |  | **Active coping** | **Acceptance** | **Planning** | **Positive reframing** | **Humor** | **Instrumental support** | **Emotional support** |
| Model 1: ADHD | β |  | -0.010 | -0.078 | -.002 | -0.053 | 0.021 | 0.084 | 0.035 |
|  | P value |  | 0.700 | 0.004 | 0.928 | 0.048 | 0.43 | 0.002 | 0.195 |
|  | CI 95% | Lower | -1.47 | -0.262- | -0.123 | -0.242 | -0.080 | 0.066 | -0.041 |
|  |  | Upper | 0.099 | -0.051 | 0.112 | -0.001 | 0.187 | 0.292 | 0.199 |
|  | R^2^ |  | 0.000 | 0.006 | 0.000 | 0.003 | 0.000 | 0.007 | 0.001 |
|  |  |  |  |  |  |  |  |  |  |
| Model 2 : ADHD +  Sociodem. | β |  | -0.001 | -0.070 | -0.036 | -0.038 | -0.047 | 0.058 | 0.025 |
|  | P value |  | 0.737 | 0.025 | 0.326 | 0.220 | 0.125 | 0.055 | 0.399 |
|  | CI 95% | Lower | -167 | -0.261 | -0.215 | -0.224 | -0.272 | -0.003 | -0.076 |
|  |  | Upper | 0.117 | -0.018 | 0.053 | 0.051 | 0.033 | 0.250 | 0.190 |
|  | R^2^ |  | 0.037 | 0.013 | 0.043 | 0.033 | 0.024 | 0.075 | 0.095 |
|  |  |  |  |  |  |  |  |  |  |
| Model 3: ADHD +  Sociodem. + clin. | β |  | -0.011 | -0.046 | -0.067 | -0.022 | -0.044 | 0.028 | 0.015 |
|  | P value |  | 0.727 | 0.139 | 0.028 | 0.470 | 0.158 | 0.355 | 0.621 |
|  | CI 95% | Lower | -0.167 | -0.214 | -0.281 | -0.190 | -0.267 | -0.067 | -0.101 |
|  |  | Upper | 0.117 | 0.030 | 0.016 | 0.088 | 0.043 | 0.185 | 0.168 |
|  | R^2^ |  | 0.041 | 0.041 | 0.087 | 0.042 | 0.025 | 0.108 | 0.098 |
|  |  |  |  |  |  |  |  |  |  |
|  |  |  | **Denial** | **Venting** | **Self-blame** | **Self-distraction** | **Substance use** | **Behavioral disengagement** | **Religion** |
| Model 1 : ADHD | β |  | 0.063 | 0.059 | 0.186 | 0.101 | 0.051 | 0.209 | -0.039 |
|  | P value |  | 0.020 | 0.029 | <0.001 | <0.001 | 0.057 | <0.001 | 0.145 |
|  | CI 95% | Lower | 0.011 | 0.014 | 0.272 | 0.108 | -0.002 | 0.235 | -0.195 |
|  |  | Upper | 0.129 | 0.253 | 0.483 | 0.344 | 0.141 | 0.390 | 0.029 |
|  | R^2^ |  | 0.004 | 0.003 | 0.034 | 0.010 | 0.003 | 0.044 | 0.002 |
|  |  |  |  |  |  |  |  |  |  |
| Model 2: ADHD +  Sociodem. | β |  | 0.043 | 0.073 | 0.065 | 0.053 | 0.051 | 0.124 | -0.025 |
|  | P value |  | 0.162 | 0.016 | 0.028 | 0.081 | 0.105 | <0.001 | 0.421 |
|  | CI 95% | Lower | -0.019 | 0.031 | 0.014 | -0.015 | -0.014 | 0.098 | -0.181 |
|  |  | Upper | 0.116 | 0.298 | 0.251 | 0.253 | 0.151 | 0.275 | 0.076 |
|  | R^2^ |  | 0.015 | 0.284 | 0.098 | 0.056 | 0.012 | 0.084 | 0.028 |
|  |  |  |  |  |  |  |  |  |  |
| Model 3: ADHD +  Sociodem. + clin. | β |  | 0.020 | 0.040 | -0.005 | 0.017 | 0.025 | 0.068 | -0.030 |
|  | P value |  | 0.519 | 0.174 | 0.841 | 0.572 | -0.047 | 0.013 | 0.333 |
|  | CI 95% | Lower | -0.045 | -0.041 | -0.115 | -0.092 | -0.114 | 0.021 | -0.194 |
|  |  | Upper | 0.089 | 0.224 | 0.093 | 0.167 | 0.151 | 0.184 | 0.066 |
|  | R^2^ |  | 0.063 | 0.120 | 0.324 | 0.139 | 0.081 | 0.243 | 0.030 |

Note: β = Standardized Coeficient β and CI 95%= Confidence interval for B. The models were run separately for each coping strategy. Model 1 included only ADHD status as a predictor. Model 2 was the same as Model 1, but also adjusted for sociodemographic data (age, gender, working status, relationship status, friends), while Model 3 was adjusted for both sociodemographic and clinical data (Covid-19 infection, self-rated health, and depressive and anxiety symptoms). *= p<0.05, **=p<0.01 ***=p<0.001
