# Supplementary material for: Coping strategies and symptoms of Adjustment Disorder among adults with Attention Deficit Hyperactivity Disorder (ADHD) during the Covid-19 pandemic
Source: PLoS One. 2024 Aug 19;19(8):e0309082. doi: 10.1371/journal.pone.0309082 (PMC11332942; doi:10.1371/journal.pone.0309082)
Supplement: S2 Table — (DOCX) [file pone.0309082.s002.docx]

**S2 Table 2 Detailed information about Associations between different coping strategies and symptoms of AjD in adults with and without ADHD.**

| **Coping strategy** | **ADHD** | | | | | | **Non-ADHD** | | | | |
| --- | --- | --- | --- | --- | --- | --- | --- | --- | --- | --- | --- |
|  | β | p-value | CI 95% | | R^2^ |  | β | p-value | CI 95% | | R^2^ |
|  |  |  | Lower | Upper |  |  |  |  | Upper | Lower |  |
| Active coping | 0.232 | <0.001 | 0.985 | 2.643 | 0.420 |  | 0.195 | <0.001 | 1.111 | 1.615 | 0.439 |
| Acceptance | 0.015 | 0.784 | -0.756 | 1.000 | 0.346 |  | -0.050 | 0.032 | -0.846 | -0.039 | 0.405 |
| Planning | 0.264 | <0.001 | 1.229 | 2.870 | 0.433 |  | 0.186 | <0.001 | 1.090 | 1.805 | 0.434 |
| Positive reframing | 0.038 | 0.501 | -0.569 | 1.161 | 0.373 |  | 0.004 | 0.853 | -0.314 | 0.379 | 0.403 |
| Humor | 0.027 | 0.629 | -0.554 | 0.915 | 0.372 |  | -0.039 | 0.088 | -0.587 | 0.041 | 0.405 |
| Instrumental support | 0.241 | <0.001 | 1.086 | 2.883 | 0.423 |  | 0.147 | <0.001 | 0.819 | 1.576 | 0.423 |
| Emotional support | 0.254 | <0.001 | 1.083 | 2.672 | 0.428 |  | 0.113 | <0.001 | 0.513 | 1.230 | 0.415 |
| Denial | 0.172 | 0.001 | 0.938 | 3.797 | 0.406 |  | 0.159 | <0.001 | 1.827 | 3.297 | 0.427 |
| Venting | 0.277 | <0.001 | 1.251 | 2.894 | 0.435 |  | 0.183 | <0.001 | 1.056 | 1.775 | 0.433 |
| Self-blame | 0.265 | <0.001 | 1.066 | 2.798 | 0.422 |  | 0.200 | <0.001 | 1.337 | 2.303 | 0.430 |
| Self-distraction | 0.298 | <0.001 | 1.530 | 3.118 | 0.454 |  | 0.222 | <0.001 | 1.356 | 2.088 | 0.445 |
| Substance use | 0,064 | 0.263 | -0.567 | 2.066 | 0.375 |  | 0.088 | <0.001 | 0.548 | 1.763 | 0.410 |
| Behavioral disengagement | 0.268 | <0.001 | 1.527 | 3.715 | 0.429 |  | 0.146 | <0.001 | 1.188 | 2.440 | 0.420 |
| Religion | 0.051 | 0.358 | -0.528 | 1.453 | 0.374 |  | 0.023 | 0.323 | -0.182 | 0.551 | 0.404 |

Note: β = Standardized Coeficient β and CI 95%= Confidence interval for B. The values are adjusted for sociodemographic and clinical data.
